# County-specific HPV epidemiology and risk factors in remote Northwestern China: A community-based study

**DOI:** 10.1016/j.aprim.2025.103417

**Published:** 2026-01-22

**Authors:** Tali Hayuehashi, Yan Wang

**Affiliations:** a School of Public Health, Xinjiang Medical University, Urumqi 830054, China; b State Key Laboratory of Pathogenesis, Prevention and Treatment of High Incidence Diseases in Central Asia, Affiliated Cancer Hospital of Xinjiang Medical University, Urumqi 830011, China

The WHO's cervical cancer elimination goal demands equitable prevention strategies, yet regions like remote Xinjiang, China, lack localized data to guide primary care interventions. While factors such as multiple sexual partners, tobacco use, and low education are well-documented HPV correlates elsewhere,^1^ their relevance in a context characterized by conservative sexual norms, limited healthcare access, and pastoralist traditions remains unverified.

To address this gap, we conducted a community-based study enrolling 3011 women aged 35–64 years in Tuoli and Huocheng counties (July–December 2024). Participants underwent HPV genotyping for 15 high-risk types (Hunan Shengxiang Biotechnology) and completed structured questionnaires (administered via REDCap) to capture demographic (education, occupation), lifestyle (secondhand smoke exposure), and clinical (urinary incontinence, abnormal vaginal discharge) factors. Stata 18.0 was used for analyses: univariate tests (*χ*^2^, Fisher's exact) screened variables with *P* < 0.25, which were then included in multivariable logistic regression to identify county-specific predictors.

HPV infection rates were 13.54% (274/2024) in Tuoli and 11.04% (109/987) in Huocheng. As shown in Table 1, multivariable analysis revealed distinct county-level associations: in Tuoli, non-exposure to secondhand smoke was linked to higher HPV risk (aOR = 2.38, 95% CI:1.10–5.18, *P* = 0.028)—a finding contrasting with global evidence on active smoking,^2^ potentially due to unmeasured confounders like local ventilation. In Huocheng, urinary incontinence was associated with HPV (aOR = 1.80, 95% CI:1.03–3.16, *P* = 0.039), aligning with U.S. research that linked low-risk HPV to lower urinary tract symptoms (OR = 1.40, 95% CI:1.01–1.95).^3^ HPV infection patterns also differed: Huocheng had more multiple infections (6.39% vs. 4.65% single), while Tuoli had more single infections (11.2% vs. 2.34% multiple; *χ*^2^, *P* < 0.001). Notably, HPV vaccination coverage was extremely low (Tuoli: 1.33%, Huocheng: 0.91%), and only 3.92% (118/3011) of women reported multiple sexual partners—reflecting the region's conservative norms. The lack of association between socioeconomic factors (education, occupation) and HPV infection (all *P* > 0.05) is consistent with evidence from other Chinese regions.^4^

**Table 1:** County-specific multivariable predictors of HPV infection in Tuoli and Huocheng (*n* = 3011).

County	Predictors	Adjusted OR [95% CI]	*P*-value
Tuoli	Exposure to secondhand smoke (No vs. Yes)	2.38 [1.10–5.18]	0.028*
	Abnormal vaginal discharge (No vs. Yes)	1.42 [0.84–2.38]	0.190

Huocheng	Urinary incontinence (No vs. Yes)	1.80 [1.03–3.16]	0.039*
	Occupation: Other jobs (vs. Jobless)	1.52 [0.94–2.46]	0.090
	Exposure to secondhand smoke (No vs. Yes)	0.75 [0.50–1.13]	0.169
	Educational attainment: High school (vs. Primary or lower)	1.34 [0.86–2.09]	0.190
	Educational attainment: College and higher (vs. Primary or lower)	1.53 [0.47–5.00]	0.481
	Occupation: Officer (vs. Jobless)	0.52 [0.14–1.87]	0.314
	Occupation: Businesswomen (vs. Jobless)	0.65 [0.25–1.69]	0.381

*Note*: OR indicates odds ratio; 95% CI, 95% confidence interval. The table presents results from the multivariable logistic regression models for each county. Statistically significant predictors (*P* < 0.05) are marked with *.

This study identifies unique county-specific HPV risk profiles in remote Northwestern China, highlighting the need for primary care interventions tailored to county-specific risks (e.g., incontinence management in Huocheng). The extremely low vaccination coverage underscores the urgency for expanded immunization programs in these underserved regions. Limitations include the cross-sectional design (limits causal inference) and single region focus, but such localized evidence is key to aligning cervical cancer prevention with primary care's preventive and community-focused mission.

## Ethical considerations

This study was approved by the Ethics Committee of the Affiliated Cancer Hospital of Xinjiang Medical University (Project Reference Code: K-2024078). All women enrolled in the study signed a paper-based informed consent form.

## Foundation

This project was supported by the 14th Five - Year Plan Distinctive Program of Public Health and Preventive Medicine in Higher Education Institutions of Xinjiang Uygur Autonomous Region, the Postgraduate Innovation Project of the School of Public Health, Xinjiang Medical University (Grant No. CXCYGW2025006), and the Research Project of the Health Commission of Xinjiang Uygur Autonomous Region (Grant No. 2025001FYJKSCZLKJZX650028739).

## Conflict of interest

The authors declare that they have no conflict of interest.
